# Mitochondrial DNA analyses found five novel mutations in idiopathic epilepsy patients

**DOI:** 10.1080/23802359.2019.1633963

**Published:** 2019-07-12

**Authors:** Cuiping You, Rui Tao, Quanping Su, Yucheng Lu, Long Wang, Shu Liu, Lifen Wang, Lijuan Wang, Fuzhong Xue, Fengyuan Che

**Affiliations:** a Department of Central Laboratory, Linyi People’s Hospital, Shandong University, Linyi, Shandong Province, China;; b Department of Neurology, Linyi People’s Hospital, Shandong University, Linyi, Shandong Province, China;; c Department of Epidemiology and Biostatistics, School of Public Health, Shandong University, Jinan, China

**Keywords:** Idiopathic epilepsy, mitochondrial DNA, variant

## Abstract

Epilepsy is a common and chronic neurological disease with a high degree of genetic heterogeneity. The etiology and pathogenesis of the disease have not been fully understood. Many studies suggested that there was a reciprocal relationship between mitochondrial dysfunction and epilepsy, but few studies focused on the mitochondrial genome (mtDNA) of the epilepsy patient which was extremely important for the mitochondrial function. In our study, we obtained complete mtDNA sequences of 27 idiopathic epilepsy patients and healthy people, and compared the sequence data with 30,000 GenBank sequences including 277 Han Chinese mtDNA sequences. We analyzed each variant that might be related to disease and examined the statistically significant variant in more than 300 patients and healthy people. Ultimately, we identified 27 variants which were reported to be associated with diseases, 4 rare variants (321T > G, 15973 T > C, 3897C > A, 12580 C > T), and a nonsynonymous variant (3571 C > T) which was predicted to be damaging. Although no variant was found to be significantly associated with epilepsy, our study provided a new insight into epilepsy study on an aspect of the mitochondrial genome.

## Introduction

Epilepsy is a common and chronic neurological disease. It results from synchronized paroxysmal repeated discharges of neurons in the central nervous system, and is characterized by spontaneously recurrent seizures (Krishnamurthy 2016). Long-term course, low cure rate and high disability rate of the disease have a profound impact on patients and society. Although numerous studies have been carried out on the pathogenesis of epilepsy over the years, the exact mechanism has not been fully elucidated. Recent years, some studies found that there was an important relationship between epilepsy and mitochondrial dysfunction, indicating that mitochondrial dysfunction might be involved in the pathogenesis of epilepsy. First, many mitochondrial diseases presented epileptic seizure, such as Leber Hereditary Optic Neuropathy (LHON) (Raule et al. 2014), Mitochondrial Myopathy, Encephalopathy, Lactic Acidosis and a Stroke-like Episode (MELAS) (Hämäläinen et al. 2013) and Myoclonic Epilepsy and Ragged Red Fibers (MERRF) (Wang et al. 2012). Majority of MELAS patients (>80%) have m.3243A > G which is located in mitochondrial tRNA leucine 1 (*MT-TL1*) (Liolitsa et al. 2003). MERRF is caused most commonly by 8344 A > G in mitochondrial tRNA lysine (*MT-TK*) (Jaksch et al. 1998). Second, some studies reported the damage of mitochondrial dysfunction in the lesion area of temporal lobe epilepsy, hippocampal sclerosis patients and animal models. These studies suggested that the disorder of energy metabolism may be related to temporal lobe epilepsy (Vielhaber et al. 2008; Fujikawa et al. 2010; Lopez‐Meraz et al. 2010).

Mitochondrion plays a critical role in producing metabolic energy in nearly all eukaryotic cells. It is also involved in many other important physiological processes, such as cell apoptosis, growth, differentiation and signal transduction. Many mtDNA mutations lead to physiological diseases, such as degenerative diseases, metabolic diseases and cancers. Therefore, it is of great significance to study the mitochondrial genome of epilepsy patients. However, few studies have been performed on the mitochondrial genomes of epilepsy patients. In the present study, we determined some mitochondrial genomes of epilepsy patients. By bioinformatic analysis, we want to find some valuable information for epilepsy study.

## Materials and methods

### Patients and DNA isolation

We recruited 200 idiopathic epilepsy patients from the Epilepsy Translational Medicine Project of Linyi People’s Hospital, Shandong, China. Phenotypic and diagnostic classification of epilepsy syndromes in the project were carried out according to 2014 ILAE Classification and Diagnostic Criteria (Fisher et al. 2014) and were reviewed by experienced epileptologists. Fifteen patients（one idiopathic partial epilepsy patient and 14 idiopathic generalized epilepsy patients）were selected for complete mtDNA sequencing. In our study, idiopathic epilepsy patients were selected as the following criteria: no any definite cause (brain trauma, stroke, tumour, intracranial infection, autoimmune diseases and so on); no lesions were found in MRI; no cognitive impairment; no other diseases (especially mitochondrial diseases); no genetic relationship with included patients. 200 healthy controls were selected from the Medical examination center of our hospital and 12 were randomly selected for complete mtDNA sequencing. This study was approved by the local Institutional Review Board and all study participants gave informed consent.

Blood sample were obtained from these patients and stored at −80 °C. Total DNA was extracted using the Genomic DNA Extraction Kit (Sangon Biotech, Shanghai, China) and stored at −20 °C prior to use.

### Mitochondrial DNA amplification and sequencing

Twenty-two pairs of PCR primers used for amplifying the complete sequences obtained from the published study (Jiang et al. 2014). PCR amplification was conducted in a 50-μL reaction mixture containing 20–40 ng DNA, 25 μL 2x Taq PCR MasterMix (TIANGEN, China), 0.8 μm of each primer. PCR conditions were 94 °C for 5 min and 30 cycles at 94 °C for 50s, 50–60 °C for 1 min, 72 °C for 1–2 min. The amplified PCR products were sent to GenScript Biotech for sequencing. Sequencing was performed on ABI 3730XL automatic sequencer.

### Data analysis

Sequences were assembled with the DNAstar 5.0 software package and aligned with MAFFT software. Mitochondrial genes were identified by BLAST. Using MitoTool (http://www.mitotool.org/index.html), all sequences were compared with the revised Cambridge Reference Sequences (rCRS, NC-012920.1) to determine the polymorphisms, variants and haplogroup status. Sequences were also compared to complete mtDNA sequences of 277 Han Chinese retrieved from GenBank. The annotation of variants and estimation of the variant pathogenicity were performed on MITOMAP which reported variants over 30000 mtDNA sequences (http://www.mitomap.org/MITOMAP) and HmtDB (http://www.hmtdb.uniba.it/hmdb/). The pathogenic potentials of nonsynonymous variants were analyzed with PolyPhen-2 (Adzhubei et al. 2013) and SIFT 4G (Vaser et al. 2016). For PolyPhen-2, the results were classified as benign, possibly damaging (score > 0.50), and probably damaging (score >0.90). For SIFT 4G, the amino acid substitution was predicted deleterious if the score was <0.05, and tolerated if the score was ≥0.05.

## Results

### mtDNA mutation analysis

The mtDNA sequences of epilepsy patients and healthy controls had been submitted to GenBank. Accession numbers, variants, length and haplotype of every sequence were shown in Supporting Information Tables 1 and 2. By alignment, we obtained 219 variable sites, 44 parsim-info sites and 175 singleton sites in 21 genes of epilepsy patients. The distribution of variable sites in each gene was shown in Supporting Information Figure 1. Compared with healthy control people in this study, with more than 30,000 GenBank and 277 Han Chinese sequences, we found 5 sites had higher frequency, especially the C16292T site which was located in the Hypervariable region 1 (HVR1) of the D-loop region. The frequency of C16292T in 277 Chinese Han sequences was 0.36%, which was significantly lower than the frequency in this study (*p* = 0.008). We examined the variant in 185 epilepsy patients and 188 healthy controls. Unfortunately, the result indicated that the variant had no statistical significance (*p* > 0.05) in large samples.

### Reported variants associated with diseases found in this study

Using MITOMAP, MitoTool and HmtDB, we found 27 variants which were associated with diseases (Table 1). 16 of these variants had very low frequencies (1.94 × 10^−4^∼0.15) in GenBank sequences, and they were not found in healthy control people in this study. By comparison, we only found 5 special variants with low frequencies associated with disease in mtDNA sequences of healthy people in this study. Most of these 27 variants were located at protein-coding genes, and were nonsynonymous substitutions. D-loop was also a sensitive region in which we found 7 variants associated with disease. Most of these diseases were mainly mental diseases and nervous system diseases, such as major depressive disorder (MDD), Alzheimer disease (AD), and Parkinson’s disease (PD).

**Table 1.** Disease-related sites in these mtDNA sequences.

Mutation Site	Mutation type	Locus	AA Position	AA Change	Disease associated*	Cases in patients	Cases in controls
150	Transition C→T	D-loop			Longevity/Cervical Carcinoma/HPV infection risk	3	4
195	T→C	D-loop			BD-associated/melanoma pts	3	0
663	A→G	12SrRNA			Coronary Atherosclerosis risk	1	0
1095	T→C	12SrRNA			SNHL	1	0
3010	G→A	16SrRNA			Cyclic Vomiting Syndrome with Migraine	5	5
3316	G→A	ND1	4	non-syn: A-T	Diabetes/LHON/PEO	1	0
3394	T→C	ND1	30	non-syn: Y=>H	LHON/Diabetes/CPT deficiency	1	0
3497	C→T	ND1	64	non-syn: A=>V	LHON	1	0
4316	A→G	tRNA-Ile			HCM with hearing loss	1	0
4833	A→G	ND2	122	non-syn: T=>A	Diabetes helper mutation; AD, PD	1	1
5178	C→A	ND2	237	non-syn: L= >M	Longevity; Extraversion MI/AMS protection; blood iron metabolism	6	5
6962	G→A	COI	353	syn:L=>L	Possible helper variant for 15927A	2	1
7706	G→A	COII	41	non-syn: A=>T	Alpers-Huttennlocher-like	1	0
8108	A→G	COII	175	non-syn: I=>V	SNHL	1	0
8414	C→T	ATPase8	17	non-syn: L=>F	Longevity	5	4
8794	C→T	ATPase6	90	non-syn: H=>Y	Exercise Endurance/Coronary Atherosclerosis risk	1	0
10454	T→C	tRNA-Arg			DEAF helper mut.	1	0
12026	A→G	ND4	423	non-syn: I=>V	DM	1	1
12361	A→G	ND5	9	non-syn: T=>A	Nonalcoholic fatty liver disease	1	0
14668	C→T	ND6	2	syn:M= >M	Depressive Disorder associated	5	4
15043	G→A	Cytb	99	syn:G= >G	MDD-associated	10	7
15662	A→G	Cytb	306	non-syn:I=>V	Complex mitochondriopathy-associated	1	0
15927	G→A	tRNA-Thr			Multiple Sclerosis/DEAF1555 increased penetrance	1	0
16093	T→C	D-loop			Cyclic Vomiting Syndrome	2	1
16183	A→C	D-loop			Melanoma patients	2	0
16189	T→C	D-loop			Diabetes/Cardiomyopathy/Endometrial cancer risk/Melanoma patients	4	2
16129	G→A	D-loop			Cyclic Vomiting Syndrome with Migraine	2	3
16192	C→T	D-loop			Melanoma patients	1	0

^*^ Information of associated diseases were retrieved from MITOMAP (http://www.mitomap.org/MITOMAP), HmtDB (http://www.hmtdb.uniba.it/hmdb/) and MitoTool (http://www.mitotool.org/).

### Rare mtDNA variants

**Figure 1.**
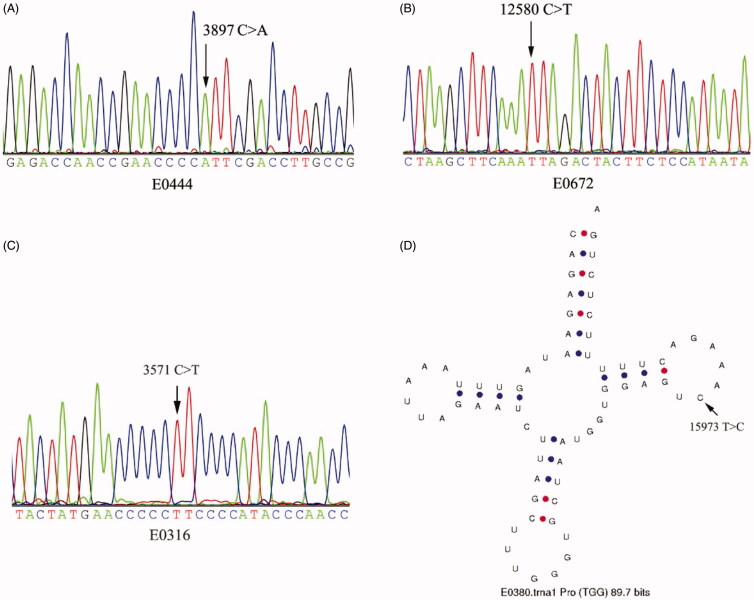
(A) mtDNA sequence of E0444 showing m.3897C > A mutation. (B) mtDNA sequence of E0672 showing m.12580 C > T mutation. (C) mtDNA sequence of E0316 showing m.3571 C > T mutation. (D) Structure of the mitochondrial tRNA^Pro^ with the mutated site indicated.

We had obtained four rare variants by MITOMAP analysis (Variant was considered rare if three or less same variants had been reported in MITOMAP.), including two sites of E0380, one site of E0444 and one site of E0672 (Supporting Information Table 3, Figure 1). These four sites had very low frequency. None of them was found in healthy people in this study. Only T321G of D-loop had a same site in 277 Chinese Han sequences. C3897A of E0444 and C12580T of E0672 were all synonymous substitutions. A15973G of E0380 were found in tRNA-Pro, and it was located at the T-loop of tRNA secondary structure.

### Nonsynonymous mtDNA mutations

Excluding the sites with frequencies higher than 6.7% (frequency of singleton site in this study) in the Han Chinese population, we detected a total of 26 nonsynonymous variants. These variants, both in GenBank and in the 277 Han Chinese population had very low frequencies. Among them, 10 sites were reported to be associated with diseases (Supporting Information Table 4). PolyPhen-2 confirmed three pathogenic sites (score > 0.900): C3571T, T12757C and A8563G. SIFT 4G confirmed six pathogenic sites (score ≤ 0.05): G7706A, T3394C, C3571T, T9128C, A4833G and A15662G. Both PolyPhen-2 and SIFT 4G predicted C3571T of E0316 to be damaging. C3571T resulted in p.Leu89Phe substitution (a branched chain amino acid changing into an aromatic amino acid) in ND1 (Supporting Information Figure 2). It didn’t occur in the mtDNA sequences of healthy controls as well.

### Mitochondrial haplogroup analysis

In haplogroup analysis, we detected three cases of haplogroup B in epilepsy patients, but not in normal people. Two cases of haplogroup N were detected in normal people while none was detected in epilepsy patients. Among the five novel loci obtained, three (321T > G, 15973 T > C and 3571 C > T) belong to haplogroup B, one (3897C > A) belongs to haplogroup M, and one (12580 C > T) belongs to haplogroup D. We compared the different haplogroup between the two groups by Fisher's exact test. The results showed that there was no significant difference (*p* > 0.05) in each haplogroup, suggesting that there is no significant difference in mitochondrial haplogroups between epilepsy patients and normal subjects. However, more accurate conclusions need to be confirmed by further research with large samples.

## Discussion

In this study, we detected 27 variants associated with diseases which were mainly mental diseases and nervous system diseases. There were 10 samples containing major depressive disorder (MDD) related sites (G15043A), and 5 samples containing depressive disorder related site (C14668T). Depressive disorder had been the most prevalent psychiatric disorder in epilepsy patients. The prevalence of depression disorder in epilepsy was as high as 30% (Josephson et al. 2017; Scott et al. 2017). Epilepsy patients are 5–20 times as likely to develop depression disorder as those without the disorder, while persons with depression disorder are more likely to develop epilepsy (Kanner 2009). Although it was not yet possible to determine whether epilepsy had a causal relationship with depression, our results showed that epilepsy patients seemed to have a higher susceptibility to depression disorder. In addition, variants associated with Cyclic Vomiting Syndrome were also numerous. There were four samples having G3010A, one sample having T16093C and one sample having G16129A. In spite of the fact that some epilepsy patients had CVS symptoms, there were no studies concerning the relationship between epilepsy and CVS. Besides, we also found three sites (G3316A, T 3394C, C3497T) of ND1 which didn’t occur in healthy control people had been reported to be associated with LHON. LHON is a typical mitochondrial disease with bilateral loss of central vision primarily due to mtDNA mutation. Sometimes, seizure was a phenotypic feature in LHON patients (Finsterer and Zarrouk-Mahjoub 2016) and members of LHON family likely exhibited seizures. In our study, variants associated with LHON belonged to sample E0672 and E0316. We did not observe symptoms of vision in these patients, indicating that these sites were not related to epilepsy, or at least not directly related to epilepsy. Moreover, we also found that the special variants associated with disease in epilepsy patients (16 variants) were far more than healthy control people (5 variants). Whether this is related to the susceptibility or threshold of epilepsy is worthy of further study.

Different from other mammalian, mitochondrial tRNAs of human have unusual secondary structures. Variants of mitochondrial tRNAs were associated with a wide range of pathologies. In MITOMAP, there were 329 tRNA variants associated with diseases, and 29 variants were confirmed to cause diseases. Frequencies of these variants in GenBank were generally low, and they were located at almost every domain of the secondary structure. T15973C of E0380 was in the T-loop of tRNA-Pro. This site was highly conserved during evolution. There was only one same variant in more than 30 thousand GenBank sequences. Many mutations in T-loop were reported to be associated with some diseases. A3288G in T-loop of mt-tRNA^Leu^ was reported to be associated with mitochondrial myopathy (Hadjigeorgiou et al. 1999). T10454C in T-loop of mt-tRNA^Arg^ was reported to be associated with insulin resistance in women with polycystic ovary syndrome (Ding et al. 2017). Mutations in mt-tRNA can destroy tRNA function directly or affect their stability (Suzuki et al. 2011). T15973C of E0308 found in our study may be also pathogenic. T321G also emerged in mtDNA of E0380. It was in Hypervariable segment 2 of D-loop, and it showed high conservation in human. Mutations of mtDNA D-loop region will lead to deficiency of mtDNA replication and transcription. However, there was no further evidence that T321G was pathogenic.

Thirteen protein-coding genes of mtDNA are essential for normal mitochondrial function. There are 300 mutations of protein-coding genes in MITOMAP associated with diseases. In this study, C3571T in ND1 of E0316 were predicted to be damaging by PolyPhen-2 and SIFT. PolyPhen-2 used a Bayesian algorithm to calculate the pathogenic potential of a nonsynonymous mutation, while SIFT used sequence homology method to classify amino acid substitutions. Deleterious amino acid substitution may affect the structure and activity of protein. Mutation C3571T led to p.Leu89Phe substitution in ND1 which encoded one subunit of OXPHOS complex I. We made a superposition analysis on the wild and mutant ND1 protein by Online software SWISS-MODEL (Schwede et al. 2003) and PyMOL (DeLano 2002). The superposition results (Supporting Information Figure 2) didn’t show noticeable difference between the wild and mutant protein structures, but that didn’t mean it was not pathogenic. Whether p.Leu89Phe substitution had an impact on the activity or assembly of complex I deserve a further study. In addition, rare variant C3897A in ND1 and C12580T in ND5 were synonymous substitutions. Although synonymous variant didn’t result in amino acid change, some studies found that they may have a role in the phenotypic manifestation of other mutation (Qin et al. 2014). On conclusion, we suggest that these novel variants have the pathogenic possibility, but further study is needed. We provided a new insight into epilepsy study on an aspect of the mitochondrial genome.

## Supplementary Material

Supplemental Material
