# Supplementary material for: Mitochondrial DNA analyses found five novel mutations in idiopathic epilepsy patients
Source: Mitochondrial DNA B Resour. 2019 Jul 12;4(2):2387–91. doi: 10.1080/23802359.2019.1633963 (PMC7707843; doi:10.1080/23802359.2019.1633963)

Supplementary table 1. Variable sites and Characteristics of the complete mtDNA sequences of epilepsy patients

| Sample | Variable sites | Length | Haplotype | Genbank number |
| --- | --- | --- | --- | --- |
| E0010 | 41, 73, 184, 195, 263, 309+C, 315+C, 489, 507, 750, 1438, 2706, 3010, 3083, 4769, 4883, 5178A, 7028, 8414, 8701, 8860, 9540, 9856, 10873, 11719, 12705, 14668, 14766, 14783, 15043, 15301, 15326, 16192, 16222, 16316, 16362 | 16570 | F2a | KY798192 |
| E0027 | 200, 215, 263, 309+C, 315+C, 318, 326, 459+C, 489, 750, 1095, 1438, 2706, 4769, 5192, 6531, 7028, 7642, 8108, 8701, 8860, 9540, 9950, 10873, 11719, 11969, 12705, 13074, 13890, 14766, 14783, 15043, 15301, 15326, 16223, 16311 | 16571 | M11b2 | KY798193 |
| E0152 | 73, 150, 263, 315+C, 489, 750, 1438, 2706, 3010, 3603, 4038, 4769, 4883, 5178A, 7028, 7706, 8414, 8701, 8745, 8860, 9540, 10356A, 10873, 11719, 12705, 14438C, 14668, 14766, 14783, 15043, 15301, 15326, 16093, 16172, 16223, 16360, 16362, 16491A, 16526T | 16569 | D41 | KY798194 |
| E0316 | 73, 150, 263, 315+C, 709, 750, 1119, 1438, 2706, 3394, 3435, 3497, 3571, 4769, 7028, 7119, 8281-8289d, 8860, 9128, 10728A, 11440, 11719, 14766, 15326, 15346, 16129, 16140, 16166, 16183d, 16189, 16217, 16274, 16335, 16519 | 16559 | B4c1b2c2 | KY798195 |
| E0380 | 73, 152, 263, 309+CC, 315+C, 321G, 411G, 460+T, 523-524d, 750, 1438, 2706, 4769, 5108, 7028, 7789, 8281-8289d, 8860, 9968, 11719, 12747+GTA, 12804, 14766, 14905, 15326, 15973, 16189, 16213, 16217, 16261, 16292, 16519 | 16564 | B4g1a | KY798196 |
| E0442 | 73, 263, 309+C, 315+C, 489, 750, 1438, 2706, 3010, 4769, 4883, 5178A, 7028, 7754, 8414, 8701, 8860, 9540, 9725, 10398, 10400, 10873, 11719, 12432, 12705, 13713, 14668, 14766, 14783, 15043, 15301, 15326, 16223, 16356, 16362 | 16570 | D4l | KY798197 |
| E0444 | 73, 146, 199, 263, 315+C, 489, 750, 1438, 2706, 3483, 3897A, 4071, 4769, 4850, 5442, 6455, 7028, 8701, 8860, 9540, 9824, 10398, 10400, 10873, 11665, 11719, 12091, 12705, 12757, 14766, 14783, 15043, 15301, 15326, 16223, 16295, 16319, 16519 | 16569 | M7c1 | KY798198 |
| E0672 | 73, 146, 263, 309+C, 315+C, 489, 750, 1438, 2706, 3010, 3316, 3606, 4316, 4769, 4883, 5178A, 7028, 8414, 8538, 8701, 8860, 9540, 9896, 10398, 10400, 10873, 11719, 12580, 12705, 14668, 14766, 14783, 14883, 15043, 15130, 15301, 15326, 16223, 16234, 16260, 16292, 16311, 16362 | 16570 | D4l | KY798199 |
| E1018 | 73, 152, 263, 309+CC, 315+C, 489, 709, 750, 1438, 2706, 4769, 4833, 5108, 5601, 7028, 8701, 8860, 9540, 9575, 10398, 10400, 10873, 11719, 11963, 12705, 13563, 14569, 14766, 14783, 15043, 15262, 15301, 15313, 15326, 16223, 16311, 16362 | 16571 | G2a'c | KY798200 |
| E1099 | 73, 103, 189, 199, 203, 204, 263, 523-524d, 709, 750, 1438, 1598, 2626, 2706, 3915, 4769, 6962, 7028, 8281-8289d, 8584, 8829, 8856, 8860, 9950, 10103, 10398, 11719, 12361, 13477, 14766, 15223, 15326, 15508, 15662, 15851, 15927, 16140, 16147, 16189, 16243, 16256, 16519 | 16557 | B5b3a | KY798201 |
| E1164 | 73, 150, 263, 750, 752, 1107, 2706, 3936G, 4769, 4883, 4973, 5178A, 5301, 6797, 7028, 8701, 8860, 9180, 9540, 10873, 11719, 11944, 12026, 12705, 14766, 14783, 15043, 15301, 15326, 16164, 16172, 16182C, 16183C, 16189, 16193+C, 16223, 16259, 16362 | 16569 | D5a2 | KY798202 |
| E1166 | 73, 235, 263, 315+C, 318, 523-524d, 663, 1438, 1736, 2706, 3826, 4248, 4769, 4824, 7028, 7352, 8563, 8659, 8794, 8860, 11536, 11719, 12705, 13260, 14599, 14766, 15326, 16124, 16223, 16290, 16319, 16519 | 16567 | A5 | KY798203 |
| E0015 | 73, 152, 195, 249d, 263, 315+C, 523-524d, 709, 750, 1438, 2706, 3970, 4769, 6392, 6599, 6962, 7028, 8860, 9053, 10310, 10454, 10609, 11719, 12406, 12882, 13759, 13928C, 14587, 14766, 15326, 16111, 16129, 16266, 16304, 16519 | 16566 | F1c1a1 | MF158628 |
| E0400 | 73, 263, 309+C, 315+C, 489, 750, 1438, 2706, 4715, 4769, 5100, 6179, 7028, 7196A, 8584, 8684, 8701, 8860, 9540, 10398, 10400, 10873, 11719, 12705, 12820, 13590, 14470, 14766, 14783, 15043, 15301, 15326, 15487T, 16086, 16134, 16172, 16184, 16223, 16298, 16319 | 16570 | M8a3a | MF158629 |
| E1295 | 73, 195, 198, 263, 315+C, 489, 750, 1438, 2706, 3010, 4769, 4883, 5004, 5178A, 7028, 8414, 8701, 8730, 8860, 9077, 9540, 10398, 10400, 10646, 10873, 11719, 12705, 13812, 14668, 14766, 14783, 15043, 15301, 15326, 16093, 16223, 16232, 16290, 16362 | 16569 | D4o2a | MF158630 |

Supplementary table 2. Variable sites and Characteristics of the complete mtDNA sequences of healthy people

| Sample | Variable sites | Length | Haplotype | Genbank number |
| --- | --- | --- | --- | --- |
| C0001 | 73, 249d, 263, 309+C, 315+C, 750, 1005, 1438, 1824, 2706, 3970, 4452, 4769, 6392, 7028, 7828, 8860, 9128, 9722, 10310, 10535, 10586, 11719, 12338, 13708, 13928C, 14766, 15326, 16203, 16254d, 16291, 16304, 16311, 16335, 16519 | 16568 | D4k | KY941897 |
| C0002 | 73, 152, 263, 309+C, 315+C, 489, 750, 1438, 1888, 2706, 3010, 3206, 4580, 4769, 4883, 5178A, 6575, 7028, 7364, 8414, 8473, 8701, 8860, 9540, 10398, 10400, 10410, 10873, 11719, 12705, 13371, 14668, 14766, 14783, 14979, 15043, 15301, 15326, 16129, 16223, 16254d, 16256, 16362, 16519 | 16569 | D4a1e | KY941898 |
| C0003 | 73, 263, 315+C, 489, 750, 1438, 2706, 3010, 3523, 4769, 4883, 5178A, 7028, 8414, 8701, 8860, 9540, 10398, 10400, 10873, 11719, 12705, 13474, 14203, 14668, 14766, 14783, 15043, 15301, 15326, 16158, 16183C, 16189, 16223, 16291, 16362, 16519 | 16569 | D4; D4l | KY941899 |
| C0004 | 73, 295, 315+C, 462, 489, 750, 1438, 1733, 2706, 3010, 4216, 4769, 4991, 5773, 7028, 8269, 8860, 10398, 11251, 11719, 12612, 13708, 14766, 15326, 15452A, 15978, 16069, 16126, 16145, 16222, 16261 | 16569 | J1b2 | KY941900 |
| C0005 | 73, 194, 263, 309+C, 315+C, 489, 523-524d, 750, 1382C, 1438, 2581, 2706, 3010, 4769, 4883, 5178A, 7028, 8020, 8414, 8701, 8860, 8964, 9296, 9540, 9824A, 9861, 10398, 10400, 10873, 11499, 11719, 12358, 12705, 14668, 14766, 14783, 15043, 15301, 15326, 16223, 16362, 16519 | 16568 | D4b2b2 | KY941901 |
| C0006 | 73, 150, 263, 309+CC, 315+C, 411G, 750, 1438, 2706, 4386, 4769, 5231, 5417, 7028, 8860, 11719, 12007, 12358, 12372, 12705, 14212, 14766, 15326, 16111, 16129, 16223, 16257A, 16261 | 16571 | N9a1 | KY941902 |
| C0007 | 73, 298, 309+C, 315+C, 489, 750, 1438, 2706, 3010, 4394, 4769, 4883, 5178A, 5231, 5655, 7028, 8414, 8701, 8860, 9540, 10398, 10400, 10873, 11059, 11719, 12372, 12705, 13022G, 13054G, 13104, 14668, 14766, 14783, 15043, 15085, 15301, 15326, 16093, 16169, 16223, 16274, 16362 | 16570 | D4g2a1 | KY941903 |
| C0008 | 73, 146, 199, 263, 309+CC, 315+C, 489, 523-524d, 750, 1438, 2706, 4071, 4769, 4850, 5442, 6455, 7028, 8701, 8860, 9449, 9540, 9824, 10398, 10400, 10873, 11665, 11719, 12091, 12705, 12810, 12960, 13106G, 14281G, 14307G, 14766, 14783, 15043, 15301, 15326, 16223, 16274, 16519 | 16569 | M7c1 | KY941904 |
| C0009 | 73, 150, 263, 309+C, 315+C, 523-524d, 750, 1438, 2706, 4386, 4769, 5231, 5417, 7028, 8860, 11719, 12007, 12358, 12372, 12705, 14381T, 14766, 14962, 15095, 15326, 16111, 16129, 16223, 16257A, 16261, 16311 | 16568 | N9a1a | KY941905 |
| C0010 | 73, 150, 263, 309+C, 315+C, 489, 523-524d, 750, 752, 1107, 2706, 3528, 4082, 4769, 4883, 5178A, 5301, 7028, 8701, 8860, 9180, 9355, 9540, 10397, 10398, 10400, 10873, 11719, 11944, 12026, 12705, 14040, 14766, 14783, 15043, 15301, 15326, 16092, 16164, 16172, 16189, 16223, 16240, 16266, 16362 | 16568 | D5a2a1b | KY941906 |
| C0011 | 73, 263, 309+C, 315+C, 489, 709, 750, 1438, 2706, 4225C, 4229A, 4769, 4833, 5108, 5601, 7028, 7600, 8701, 8860, 9377, 9540, 9575, 10398, 10400, 10873, 11719, 12705, 13563, 13928C, 14200, 14569, 14766, 14783, 15043, 15301, 15326, 16223, 16274, 16278, 16362 | 16570 | G2a1 | KY941907 |
| C0012 | 73, 146, 150, 249d, 263, 309+C, 315+C, 523-524d, 750, 1438, 2706, 3970, 4086, 4769, 6392, 6962, 7028, 8149, 8860, 9053, 9548, 10310, 10609, 11719, 12406, 12882, 13759, 13928C, 14766, 15326, 16108, 16162, 16172, 16304, 16519 | 16567 | F1a1a | KY941908 |

Supplementary Table 3. Rare mtDNA variants discovered in patients with epilepsy

| Mutation Site | samples | Mutation type | Locus | AA Change | GB Sequences^1^ | Chinese Han Sequences^2^ |
| --- | --- | --- | --- | --- | --- | --- |
| 321 | E0380 | T→G | D-loop |  | 3 | 1 |
| 15973 | E0380 | A→G | tRNA-Pro |  | 1 | 0 |
| 3897 | E0444 | C→A | ND1 | [syn: P-P](https://www.mitomap.org/bin/view/MITOMAP/HumanMitoCode) | 2 | 0 |
| 12580 | E0672 | C→T | ND5 | [syn: L-L](https://www.mitomap.org/bin/view/MITOMAP/HumanMitoCode) | 1 | 0 |

^1^Number of sequences among 32059 full length GenBank sequences and 66676 control regions in MITOMAP database.

^2^ Number of sequences among 277 Chinese Han sequences.

Supplementary Table 4. Nonsynonymous mtDNA mutations in epilepsy patients

| samples | Mutation Site | Mutation type | Locus | AA Change | Frequency in GB Sequences (%) | Frequency in 277 Chinese Han Sequences | Polyphen  score^1^ | SIFT score^2^ |
| --- | --- | --- | --- | --- | --- | --- | --- | --- |
| E0027 | 8108 | A→G | COII | [I175V](https://www.mitomap.org/bin/view/MITOMAP/HumanMitoCode) | 0.16 | 1.81 | 0.005 | 0.77 |
|  | 11969 | G→A | ND4 | [A404T](https://www.mitomap.org/bin/view/MITOMAP/HumanMitoCode) | 1.51 | 2.52 | 0.003 | 0.13 |
| E0152 | 7706 | G→A | COII | [A41T](https://www.mitomap.org/bin/view/MITOMAP/HumanMitoCode) | 0.02 | 0.00 | 0.338 | 0.03 |
| E0316 | 3394 | T→C | ND1 | [Y30H](https://www.mitomap.org/bin/view/MITOMAP/HumanMitoCode) | 1.28 | 1.81 | 0.080 | 0.05 |
|  | 3497 | C→T | ND1 | [A64V](https://www.mitomap.org/bin/view/MITOMAP/HumanMitoCode) | 0.36 | 1.81 | 0.009 | 0.51 |
|  | 3571 | C→T | ND1 | [L89F](https://www.mitomap.org/bin/view/MITOMAP/HumanMitoCode) | 0.24 | 1.81 | 0.954 | 0.02 |
|  | 7119 | G→A | COI | [D406N](https://www.mitomap.org/bin/view/MITOMAP/HumanMitoCode) | 0.09 | 0.36 | 0.004 | 1.00 |
|  | 9128 | T→C | ATP6 | [I201T](https://www.mitomap.org/bin/view/MITOMAP/HumanMitoCode) | 0.20 | 2.17 | 0.003 | 0.02 |
| E0442 | 7754 | G→A | COII | [D57N](https://www.mitomap.org/bin/view/MITOMAP/HumanMitoCode) | 0.06 | 0.00 | 0.004 | 0.17 |
| E0444 | 12757 | T→C | ND5 | [F141L](https://www.mitomap.org/bin/view/MITOMAP/HumanMitoCode) | 0.04 | 0.00 | 0.990 | 0.10 |
| E0672 | 3316 | G→A | ND1 | [A4T](https://www.mitomap.org/bin/view/MITOMAP/HumanMitoCode) | 0.95 | 1.81 | 0.001 | 0.39 |
|  | 14883 | C→T | Cytb | [T46I](https://www.mitomap.org/bin/view/MITOMAP/HumanMitoCode) | 0.04 | 0.00 | 0.001 | 0.18 |
| E1018 | 4833 | A→G | ND2 | [T122A](https://www.mitomap.org/bin/view/MITOMAP/HumanMitoCode) | 0.82 | 3.25 | 0.433 | 0.01 |
|  | 11963 | G→A | ND4 | [V402I](https://www.mitomap.org/bin/view/MITOMAP/HumanMitoCode) | 0.2 | 0.72 | 0.000 | 1.00 |
| E1099 | 12361 | A→G | ND5 | [T9A](https://www.mitomap.org/bin/view/MITOMAP/HumanMitoCode) | 0.59 | 0.72 | unknown | 0.44 |
|  | 13477 | G→A | ND5 | [A381T](https://www.mitomap.org/bin/view/MITOMAP/HumanMitoCode) | 0.06 | 0.36 | 0.007 | 1.00 |
|  | 15851 | A→G | Cytb | [I369V](https://www.mitomap.org/bin/view/MITOMAP/HumanMitoCode) | 0.36 | 0.72 | 0.003 | 0.61 |
|  | 15662 | A→G | Cytb | [I306V](https://www.mitomap.org/bin/view/MITOMAP/HumanMitoCode) | 0.38 | 0.72 | 0.002 | 0.05 |
| E1164 | 12026 | A→G | ND4 | [I423V](https://www.mitomap.org/bin/view/MITOMAP/HumanMitoCode) | 0.51 | 2.89 | 0.010 | 0.12 |
| E1166 | 4824 | A→G | ND2 | [T119A](https://www.mitomap.org/bin/view/MITOMAP/HumanMitoCode) | 3.02 | 4.70 | 0.318 | 0.16 |
|  | 8563 | A→G | ATP6 | [T13A](https://www.mitomap.org/bin/view/MITOMAP/HumanMitoCode) | 0.47 | 0.72 | 0.988 | 0.22 |
|  | 8659 | A→G | ATP6 | [T45A](https://www.mitomap.org/bin/view/MITOMAP/HumanMitoCode) | 0.17 | 0.72 | 0.005 | 0.17 |
|  | 8794 | C→T | ATP6 | [H90Y](https://www.mitomap.org/bin/view/MITOMAP/HumanMitoCode) | 2.94 | 3.61 | 0.003 | 1.00 |
| E0400 | 8684 | C→T | ATP6 | [T53I](https://www.mitomap.org/bin/view/MITOMAP/HumanMitoCode) | 0.51 | 2.53 | 0.005 | 1.00 |
|  | 12820 | G→A | ND5 | [A162T](https://www.mitomap.org/bin/view/MITOMAP/HumanMitoCode) | 0.02 | 0.00 | 0.001 | 1.00 |
| E1295 | 9077 | T→C | ATP6 | [I184T](https://www.mitomap.org/bin/view/MITOMAP/HumanMitoCode) | 0.16 | 0.00 | 0.003 | 0.12 |

^1^<http://genetics.bwh.harvard.edu/pph2/> Pathogenicity prediction levels: 0 (lowest reliable) to 1.0 (most reliable).

^2^<http://sift.bii.a-star.edu.sg/> Damaging amino acid substitutions≤0.05.

Supplementary Figure 1. The distribution of variable sites in each gene


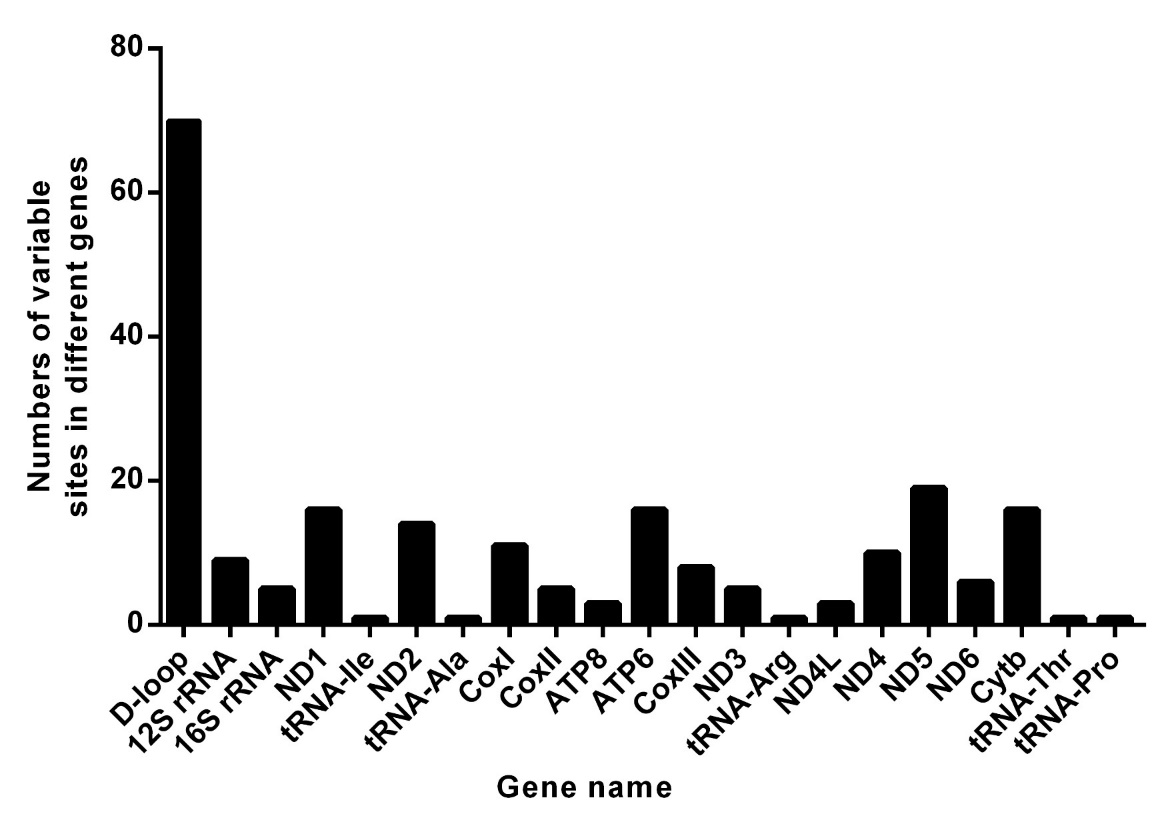


Supplementary Figure 2. (A) The 3D structure of ND1 protein in complex I of oxidative phosphorylation system. (B) The superimposed 3D structure of normal and C3571T mutant ND1 protein.


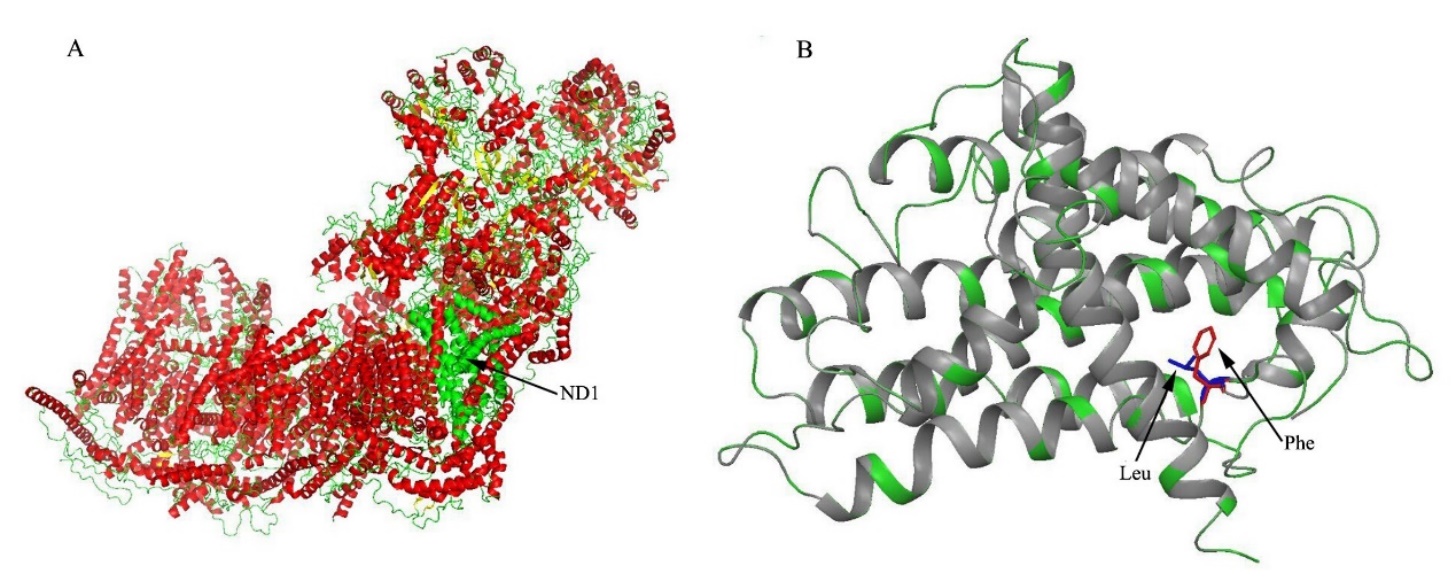

Supplement: Supplemental Material [file TMDN_A_1633963_SM3556.zip › Fengyuan et al supplemental content.docx]
